# Molecular investigation and phylogeny of species of the *Anaplasmataceae* infecting animals and ticks in Senegal

**DOI:** 10.1186/s13071-019-3742-y

**Published:** 2019-10-22

**Authors:** Mustapha Dahmani, Bernard Davoust, Masse Sambou, Hubert Bassene, Pierre Scandola, Tinhinene Ameur, Didier Raoult, Florence Fenollar, Oleg Mediannikov

**Affiliations:** 1 Microbes, Evolution, Phylogeny and Infection (MEPHI), UMR Aix-Marseille University, IRD, APHM, IHU Méditerranée Infection, 19-21, Bd Jean Moulin, 13005 Marseille, France; 2 IHU Méditerranée Infection, 19-21, Bd Jean Moulin, 13005 Marseille, France; 3 Department of Biological Sciences, Old Dominion University, Norfolk, VA USA; 4 Vectors-Tropical and Mediterranean Infections (VITROME), Campus International UCAD-IRD, Dakar, Sénégal; 5 VITROME, UMR Aix-Marseille University, IRD, SSA, APHM, IHU Méditerranée Infection, 19-21, Bd Jean Moulin, 13005 Marseille, France

**Keywords:** *Anaplasmataceae*, PCR, Ticks, Horse, Dog, Donkey, Ruminants, Senegal

## Abstract

**Background:**

Our study aimed to assess the diversity of the species of *Anaplasmataceae* in Senegal that infect animals and ticks in three areas: near Keur Momar Sarr (northern region), Dielmo and Diop (Sine Saloum, central region of Senegal), and in Casamance (southern region of Senegal).

**Methods:**

A total of 204 ticks and 433 blood samples were collected from ruminants, horses, donkeys and dogs. Ticks were identified morphologically and by molecular characterization targeting the *12S* rRNA gene. Molecular characterization of species of *Anaplasmataceae* infecting Senegalese ticks and animals was conducted using the *23S* rRNA, *16S* rRNA, *rpoB* and *groEL* genes.

**Results:**

Ticks were identified as *Rhipicephalus evertsi evertsi* (84.3%), *Hyalomma rufipes* (8.3%), *Hyalomma impeltatum* (4.9%), *R. bursa* (1.5%) and *R. muhsamae* (0.9%). The overall prevalence of *Anaplasmataceae* infection in ticks was 0.9%, whereas 41.1% of the sampled animals were found infected by one of the species belonging to this family. We identified the pathogen *Anaplasma ovis* in 55.9% of sheep, *A. marginale* and *A. centrale* in 19.4% and 8.1%, respectively, of cattle, as well as a putative new species of *Anaplasmataceae.* Two *Anaplasma* species commonly infecting ruminants were identified. *Anaplasma* cf. *platys*, closely related to *A. platys* was identified in 19.8% of sheep, 27.7% of goats and 22.6% of cattle, whereas a putative new species, named here provisionally “*Candidatus* Anaplasma africae”, was identified in 3.7% of sheep, 10.3% of goats and 8.1% of cattle. *Ehrlichia canis* and *Anaplasma platys* were identified only from dogs sampled in the Keur Momar Sarr area. *Ehrlichia canis* was identified in 18.8% of dogs and two *R. e. evertsi* ticks removed from the same sheep. *Anaplasma platys* was identified in 15.6% of dogs. Neither of the dogs sampled from Casamance region nor the horses and donkeys sampled from Keur Momar Sarr area were found infected by an *Anaplasmataceae* species.

**Conclusions:**

This study presents a summary of *Anaplasmataceae* species that infect animals and ticks in three areas from the northern, central and southern regions of Senegal. To our knowledge, our findings demonstrate for the first time the presence of multiple *Anaplasmataceae* species that infect ticks and domestic animals in Senegal. We recorded two potentially new species commonly infecting ruminants named here provisionally as *Anaplasma* cf*. platys* and “*Candidatus* Anaplasma africae”. However, *E. canis* was the only species identified and amplified from ticks. None of the other *Anaplasmataceae* species identified in animals were identified in the tick species collected from animals.

## Background

A member of the order Rickettsiales, the family *Anaplasmataceae* contains the zoonotic intracellular alpha-proteobacteria of the genera *Anaplasma*, *Ehrlichia*, *Neoehrlichia* and *Neorickettsia* [1]. These vector-borne bacteria are transmitted mainly by ixodid ticks (*Anaplasma*, *Ehrlichia* and *Neoehrlichia*) whereas *Neorickettsia* are intracellular endosymbionts of a diverse group of the Digenea (Platyhelminthes: Trematoda) [2]. In ticks, transmission of *Anaplasma* and *Ehrlichia* species occurs transtadially but not transovarially; therefore, every tick generation must obtain infection by feeding on reservoir hosts [3]. *Anaplasma* and *Ehrlichia* are able to cause a persistent infection in the vertebrate hosts, which allows them to be reservoirs of the infection [4, 5]. The nature of the infection cycle and the virulence of different strains of *Anaplasma* and *Ehrlichia* depend on the susceptibility of the infected vertebrate hosts and the availability and abundance of ixodid tick vectors largely interconnected in an epidemiological network [6, 7]. The persistent infection induced by *Anaplasma* or *Ehrlichia* can cause death in animals due to co-infection by *Staphylococcus aureus* or *Mannheimia/Bibersteinia*, “pasteurellosis” and other opportunistic diseases [8]. Animals are variably susceptible to the different strains of *Anaplasma* and *Ehrlichia*. For example, the American *Anaplasma phagocytophilum* strain human-active (Ap-ha) and variant 1 (Ap-v1) [9] seem to be less pathogenic to animals and fail to induce disease or marked bacteremia [10]. However, the European *A. phagocytophilum* strains are pathogens for cattle, sheep, goats, dogs and cats [11]. Bovine anaplasmosis caused by *A. marginale* is a worldwide reported infection. It results in the development of mild to severe anemia [12]. *Anaplasma marginale* and *Babesia* spp. together are responsible for economic losses reaching 22 and 57 million USD in Australia and India, respectively [12, 13]. *Anaplasma ovis* is a neglected agent of sheep and goat anaplasmosis due to the usually subclinical course of the disease [14]. Research in the last decade has further elucidated the nature of the syndrome caused by anaplasmosis in the infected host, the importance of animals as a reservoir of this bacteria, and the zoonotic potential of some *Anaplasma* spp. [5, 15, 16].

In Africa, the prevalence of *Anaplasmataceae* infection and extent to which livestock productivity has been affected remain poorly understood. These bacteria were recorded in many countries in southern Africa but few studies have been conducted in West Africa [17]. To better understand the epidemiological significance of the *Anaplasmataceae* infection in animals, it is necessary to include in the analyses multiple samples from different mammalian hosts and vectors.

The current reported statistics about livestock numbers, distribution and economic importance are difficult to evaluate in Senegal. Our main objective was to provide updated information about the local spread and epidemiology of infectious diseases in animals and ticks in three areas near Keur Momar Sarr (northern region of Senegal), Dielmo and Diop (Sine Saloum, central region of Senegal) and Casamance (southern region of Senegal). The aim of this study was to provide a detailed overview regarding the presence and the prevalence of *Anaplasmataceae* species infecting and currently circulating in and between cattle, sheep, goats, horses, donkeys, dogs and ticks in this region, to evaluate the genetic diversity of these bacteria and, finally, to carry out their phylogenetic characterization.

## Methods

### Period, study areas and collection of ticks and blood samples

**Fig. 1:**
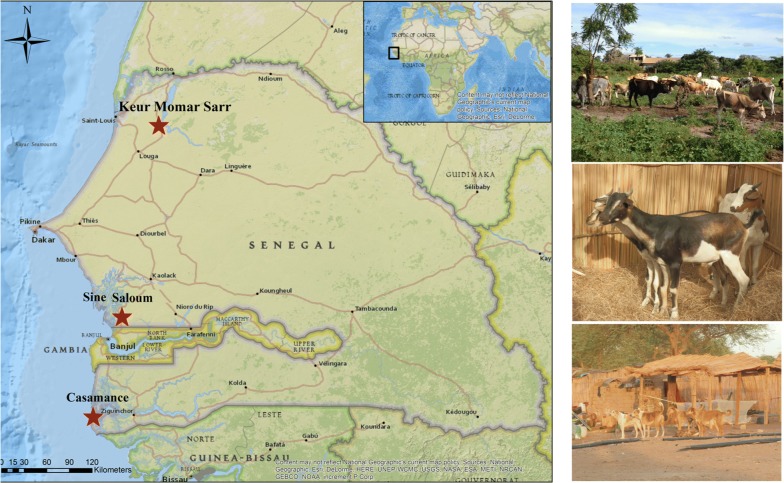
Location of the study

All animals and ticks were sampled and collected in June 2013 and June 2014 from three Senegalese areas (Fig. 1): (i) the northern region near Keur Momar Sarr (15°55′0.0012″N, 15°58′0.0012″W): Gankette Balla (15°58′50.6”N, 15°55′42.6”W), Loboudou (15°57′10.98″N, 15°55′11.8668″W) and Ndimb (16°2′56.958″N, 16°0′10.5876″W); (ii) Sine Saloum (central region of Senegal near Gambia): Dielmo (13°43′0.0012″N, 16°23′60″W) and Diop (13°40′59.9988″N, 16°21′59.976″W); and (iii) Casamance (southern region): Oussouye (12°29′13.8768″N, 16°32′52.8288″W). Animals were examined with the assistance of their owners. Ticks and blood samples were collected by a veterinarian. Overall, in 2013, 47 blood samples from 47 cattle were sampled in Dielmo and Diop, and 78 dog blood samples were collected in Casamance. In 2014, other EDTA blood samples and ticks were also collected from 136 sheep, 29 goats, 15 cattle, 64 horses, 29 donkeys and 64 dogs in the Keur Momar Sarr area (Table 1). Two hundred four adult ticks were manually collected from animals. Blood samples and ticks (stored in 70% ethanol) were transported to the laboratory of the IHU Méditerranée Infection, Marseille (France). Upon arrival, all blood samples were stored at − 80 °C. Ticks in ethanol were stored at room temperature.

**Table 1:** Primers and probes used in this study

Species	Target gene	Primer and probe	Sequence (5′–3′)	T (°C)	Reference
qPCR					
*Anaplasmataceae*	*23S* rRNA	TtAna-F	TGACAGCGTACCTTTTGCAT	60	[23, 24]
		TtAna-R	GTAACAGGTTCGGTCCTCCA		
		TtAna-S	FAM-GGATTAGACCCGAAACCAAG-TAMRA		
Conventional PCR					
*Anaplasma* spp.	*23S* rRNA	Ana23S-212f	GTTGAAAARACTGATGGTATGCA	55	[23, 24]
		Ana23S-753r	TGCAAAAGGTACGCTGTCAC		
	*16S* rRNA	AENW-16S-F1	GCAGACGGGTGMGTAAYG	50	[73]
		AENW-16S-R	GTGCCAGCAGCCGCGGTAAT		
		AENW-16S-F2 (seq.)	GTGCCAGCAGCCGCGGTAAT		
	*rpoB*	Ana-rpoBF	GCTGTTCCTAGGCTYTCTTACGCGA	55	[22]
		Ana-rpoBR	AATCRAGCCAVGAGCCCCTRTAWGG		
*Ehrlichia* spp.	*groEL*	Ehr-*groEL*-F	GTTGAAAARACTGATGGTATGCA	50	[22]
		Ehr-*groEL*-R	ACACGRTCTTTACGYTCYTTAAC		
Ticks	*12S* rDNA	T1B	AAACTAGGATTAGATACCCT	51	[21]
		T2A	AATGAGAGCGACGGGCGATGT		

*Abbreviation*: T, annealing temperature; seq., sequencing

Ticks were identified morphologically under a binocular microscope. Ticks were classified by family, genus and species using available taxonomic keys and morphometric tables [18–20]. In addition, to confirm the morphological identification, three ticks from each tick species and all ticks that were not identified or only identified at the family level, including engorged females and damaged ticks, were subjected to a molecular identification using primers targeting the mitochondrial *12S* rDNA as described previously [21].

### DNA extraction

DNA extraction was performed on a Bio Robot EZ1 (Qiagen, Courtaboeuf, France) using a commercial EZ1 DNA Tissue Kit (Qiagen) according to the manufacturer’s instructions. DNA was extracted from 200 µl of blood from all the animal samples. Ticks were recovered from ethanol, rinsed with distilled water and dried on sterile filter paper in a laminar-flow hood. Each tick was cut in half lengthways (the blades were discarded after each tick was cut). DNA was individually extracted from one-half, and the remaining tick halves were frozen at − 80 °C for subsequent studies as previously described [22].

### PCR amplification

DNA samples from the selected tick species were subjected to PCR amplification using primers targeting the 360-bp long fragment of the mitochondrial *12S* rDNA gene. In order to investigate the presence of *Anaplasmataceae* in Senegalese ticks and in domestic animal blood samples, DNA samples were initially screened by a qPCR targeting the *23S* rRNA gene. This qPCR determined that most bacteria belonged to the family *Anaplasmataceae* [23]. Then, all positive samples were subjected to a conventional PCR using primers that amplify a 485-bp long fragment of the *23S* rRNA gene, as previously described [24]. In order to mine deeper into the identity of selected *Anaplasmataceae* species in domestic animals or ticks, one or more DNA samples representative of each *Anaplasmataceae* species previously identified by the *23S* rRNA gene were amplified using primers specifically targeting the *16S* rRNA gene for the family *Anaplasmataceae* (828 bp), the RNA polymerase subunit beta (*rpoB*) gene for the genus *Anaplasma* (525 bp), and specific primers targeting the heat-shock protein (*groEL*) gene for the genus *Ehrlichia* (590 bp) (Table 2).

**Table 2:** Summary of the number of animal and ticks sampled and overall results reported in the present study

Animals	No. examined	Region	Species amplified	No. of infected animals (%)	No. of animals infested by ticks	Tick species	No. of ticks examined	No. of infected ticks (%	Species amplified
Sheep	136	Keur Momar Sarr	*Anaplasma ovis*	76 (55.9)	15	*Rhipicephalus evertsi evertsi*	48	2 (4.2)	*Ehrlichia canis*
			*Anaplasma* cf*. platys*	27 (19.8)					
			“*Ca.* Anaplasma africae”	5 (3.7)					
Cattle	47	Sine Saloum	*A. marginale*	10 (21.3)		Not found			
2013			*A. centrale*	3 (6.4)					
			*Anaplasma* cf*. platys*	12 (25.5)					
			“*Ca.* Anaplasma africae”	4 (8.5)					
2014	15	Keur Momar Sarr	*A. marginale*	2 (13.3)	3	*R. e. evertsi*	5	na	
			*A. centrale*	3 (20.0)		*Hyalomma rufipes*	4	na	
			*Anaplasma* cf*. platys*	2 (13.3)					
			“*Ca.* Anaplasma africae”	1 (6.7)					
Goats	29	Keur Momar Sarr	*Anaplasma* cf*. platys*	8 (27.7)		Not found			
			“*Ca.* Anaplasma africae”	3 (10.3)					
Equines	64	Keur Momar Sarr	na		2 donkeys	*R. e. evertsi*	6	na	
					26 horses	*R. e. evertsi*	113	na	
						*Hy. impeltatum*	10		
						*Hy. rufipes*	13	na	
						*R. bursa*	3	na	
Dogs	64	Keur Momar Sarr	*A. platys*	10 (15.6)	2	*R. muhsamae*	2	na	
			*E. canis*	12 (18.8)					
	78	Casamance		0 (0)		Not found			
Total	433			178 (41.1)	48		204	2 (0.9)	

PCR amplifications were performed as described previously [23, 24]. The real-time PCR assays were performed on a CFX96 Touch detection system (Bio-Rad, Marnes-la-Coquette, France) using Takyon Master Mix under the conditions suggested by the manufacturer. The conventional PCRs were performed in automated DNA thermal cyclers (GeneAmp PCR Systems, Applied Biosystems, Courtaboeuf, France). The amplification reactions were performed under the following conditions: an initial denaturation step at 95 °C for 15 min, followed by 40 cycles of 1 min denaturation at 95 °C, 1 min annealing at a corresponding temperature (Table 2) and 1 min extension at 72 °C. A final extension cycle at 72 °C for 7 min was performed, and the reactions were cooled to 15 °C. Distilled water and DNA of *A. phagocytophilum* obtained from HL60 infected cell line maintained in our laboratory, and *Ehrlichia canis* obtained from old DNA extracted from infected dogʼs blood sampled in Algeria from our previous study [24] were used in each test as negative and positive controls, respectively. The amplification products were visualized on 1.5% agarose gels stained with ethidium bromide and examined by UV transillumination. A DNA molecular weight marker (marker VI, Boehringer, Mannheim, Germany) was used to estimate the sizes of the products.

### Sequencing and phylogenetic analyses

Sequencing analyses were performed on an Applied Biosystems 3130xl Genetic Analyzer (Thermo Fisher Scientific, Les Ulis, France) using the DNA sequencing Big Dye Terminator Kit (Perkin-Elmer, Hamburg, Germany) as described by the manufacturer. The obtained sequences were assembled using ChromasPro v.1.7 software (Technelysium Pty Ltd., Tewantin, Australia). The sequences of primers were removed and the newly generated sequences were aligned with other tick or *Anaplasmataceae* species sequences available on GenBank using CLUSTAL W implemented in BioEdit v.3 [25]. The sequences of *12S* rDNA from ticks and the sequences of the *23S* rRNA, *rpoB* and *groEL* genes were first aligned individually; gaps and missing data were eliminated. Then, the sequence alignments of the *23S* rRNA, *16S* rRNA with *rpoB* genes for *Anaplasmataceae* species, and *23S* rRNA, *16S* rRNA with *groEL* genes for *Ehrlichia* species were concatenated for phylogenetic tree construction. Phylogenetic relationships and molecular evolution were inferred using the maximum likelihood method implemented in MEGA7 [26], with the complete deletion option, based on the Hasegawa–Kishino–Yano model. A discrete gamma distribution was used to model evolutionary rate differences among sites. Initial trees for the heuristic search were obtained automatically by applying the Neighbor-Join and BioNJ algorithms to a matrix of pair wise distances estimated using the maximum composite likelihood approach. Statistical support for internal branches of the trees was evaluated by bootstrapping with 1000 iterations.

## Results

### Ticks morphological and molecular identification

**Fig. 2:**
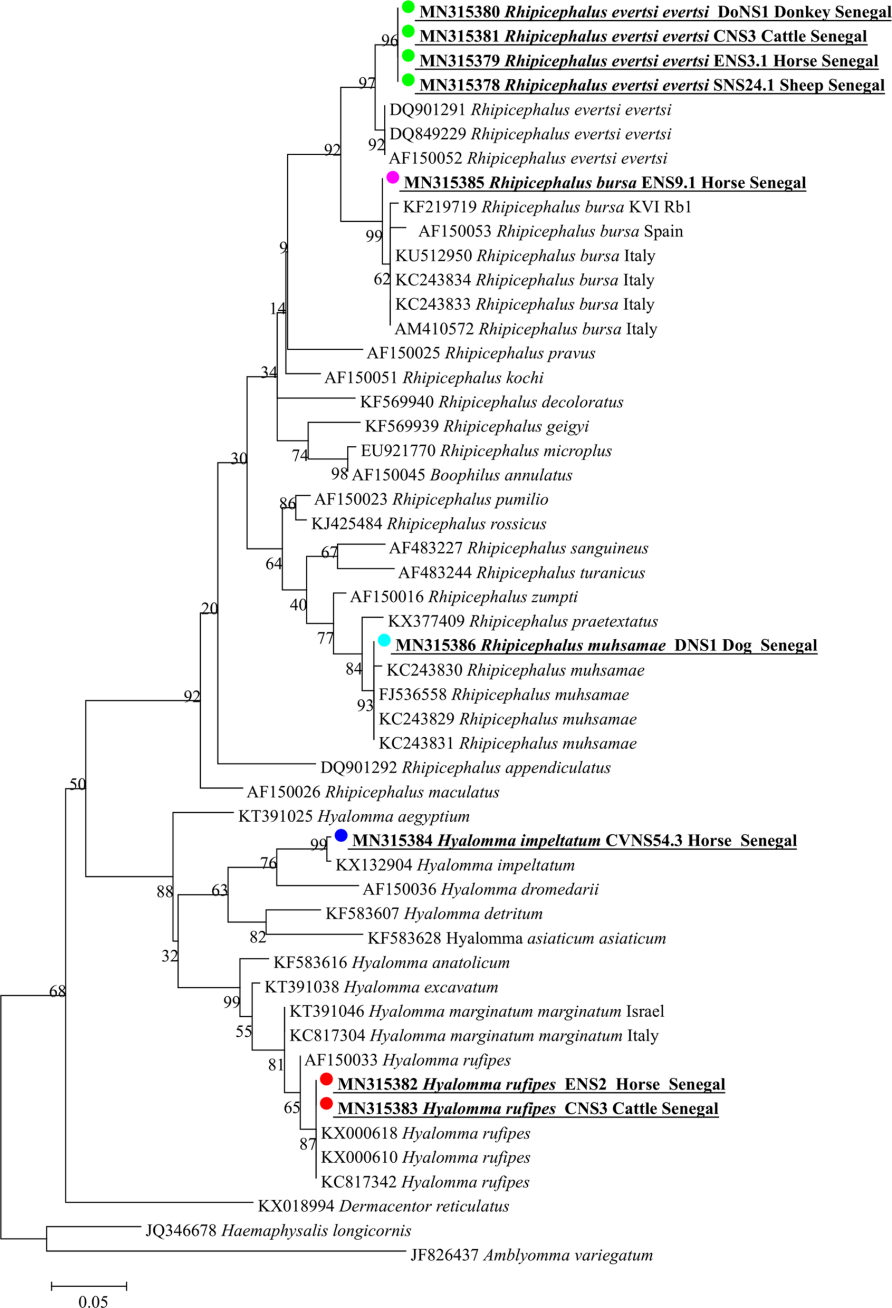
Phylogenetic tree showing the position of *R. evertsi evertsi*, *Hy. rufipes*, *Hy. impeltatum*, *R. bursa* and *R. muhsamae* compared to other tick species. Evolutionary analyses were conducted using MEGA7 [26]. The sequences of the *12S* rDNA amplified in this study with other *12S* rDNA tick sequences available on GenBank were aligned using CLUSTAL W implemented on BioEdit v.3 [25] (there were 262 positions in the final dataset). The evolutionary history was inferred by using the maximum likelihood method based on the Hasegawa–Kishino–Yano model. The percentage of trees in which the associated taxa clustered together is shown next to the branches. Initial tree for the heuristic search was obtained automatically by applying Neighbor-Join and BioNJ algorithms to a matrix of pairwise distances estimated using the maximum composite likelihood approach and then selecting the topology with superior log likelihood value. Statistical support for internal branches of the trees was evaluated by bootstrapping with 1000 iterations. A discrete gamma distribution was used to model evolutionary rate differences among sites [2 categories (+G, parameter = 0.4726)]. The analysis involved 52 nucleotide sequences. All positions containing gaps and missing data were excluded. The tree is drawn to scale, with branch lengths measured in the number of substitutions per site. The scale-bar represents a 5% nucleotide sequence divergence

All ticks were collected in the Keur Momar Sarr area. A total of 204 ticks were collected; of these, 64.2% (131) were male and 35.8% (73) were female. One hundred thirty-nine ticks were removed from 26 horses, 46 ticks from 15 sheep, 9 ticks from 3 cattle, 6 ticks from 2 donkeys and 2 ticks from 2 dogs (Table 1). One to 16 ticks were collected from each animal. One hundred seventy-seven ticks were morphologically identified as follows: 154 *Rhipicephalus evertsi evertsi* (75.5%), 14 *Hyalomma rufipes* (6.9%) and 9 *Hyalomma impeltatum* (4.4%). Twenty-seven ticks were not identified at species level. These ticks included fully-engorged and damaged female ticks (*n* = 25) and two ticks removed from two dogs identified at the *Rhipicephalus* sp. level. These ticks were subject to molecular characterization. In addition to ticks not identified morphologically, at least three samples of *R. e. evertsi*, *Hy. impeltatum*, and *Hy. rufipes* identified morphologically were subjected to further molecular identification. Finally, 47 ticks were used for the *12S* rRNA gene amplification. Each sequence generated from each amplicon and belonging to the same species were aligned individually using CLUSTAL W; then, gaps and single-nucleotide polymorphisms (SNP) were corrected. The 25 previously unidentified engorged females were identified as follows: 18 *Rhipicephalus evertsi evertsi*, 1 *Hy. impeltatum*, 3 *Hy. rufipes* and 3 *R. bursa*. The two ticks taken from dogs were identified as *R. muhsamae.* From the 20 remaining ticks identified morphologically that were subjected to molecular characterization, we did not find any discordance between molecular and morphological identification. The sequences of *R. e. evertsi* showed 98% identity with *R. e. evertsi* collected in Zimbabwe (GenBank: AF150052) and Zambia (GenBank: DQ901291–DQ849229). The sequences of *Hy. impeltatum* showed 99% identity with *Hy. impeltatum* collected in Niger (GenBank: KX132904). *Hyalomma rufipes* showed 100% identity with the sequences of *Hy. rufipes* collected in France (GenBank: KX000618–KX000610) and Italy (GenBank: KC817342). The sequences of *R. bursa* showed 99% identity with the sequences of *R. bursa* collected in Italy (GenBank: AM410572, KC243833, KC243834 and KU512950) and Spain (GenBank: AF150053). Finally, the sequences of *R. muhsamae* showed 100% identity with *R. muhsamae* collected in Nigeria (GenBank: KC243829). The phylogenetic tree comparing the sequences of *12S* rRNA gene amplified from our ticks to other sequences of the same gene available in GenBank is presented in Fig. 2.

All tick species collected are listed in Table 1. *Rhipicephalus evertsi evertsi* were the most commonly collected ticks in all animals (172, 84.3%) except from dogs. *Hyalomma rufipes* (17, 8.3%) were collected from four cattle and 13 horses. *Hyalomma impeltatum* (10, 4.9%) and *R. bursa* (3, 1.5%) were both collected from three different horses. Finally, *R. muhsamae* (2, 0.9%) were removed only from dogs (Table 1). Occurrence of more than one tick species on a single animal was observed: 2 cattle were found infested with *R. e. evertsi* and *Hy. rufipes*; co-occurrence of *R. e. evertsi* with *Hy. rufipes*, and *R. e. evertsi* with *R. bursa* was observed in 5 and 3 horses, respectively; and one horse was found infested by three tick species, *R. e. evertsi*, *Hy. rufipes* and *Hy. impeltatum.*

### *Anaplasmataceae* species screening and sequencing

All results are summarized in Table 2. The 637 DNA samples extracted from ticks and animal blood were first screened using qPCR targeting the *23S* RNA gene of the *Anaplasmataceae*. In ticks, only two *Rh. e. evertsi* (0.9%) collected from the same sheep were found positive. In animals, 108/136 sheep (79.4%), 11/29 goats (37.9%), 38/62 cattle (61.3%) and 22/142 dogs (15.5%) were found positive. Sheep, goats and dogs positive for *Anaplasmataceae* in the qPCR were all from Keur Momar Sarr. For cattle, 29 positives were from the Sine Saloum in the central region of Senegal (29/47; 61.7%) and eight were from the Keur Momar Sarr in the northern region of Senegal (8/15; 53.3%).

Using primers that amplify a 485-bp long fragment of the *23S* rRNA gene, all the *Anaplasmataceae*-positive samples identified in the qPCR were amplified and sequenced. The sequences generated from each amplicon belonging to the same species were aligned individually using CLUSTAL W; then, gaps and SNP were corrected. *Ehrlichia canis* was amplified from 2 *R. e. evertsi* and showed 99% identity with *E. canis* strain Jack (GenBank: NR076375). *Anaplasma ovis* was identified exclusively in sheep. The sequences showed 100% with *A. ovis* genotype KMND Niayes 14 (GenBank: KM021411). *Anaplasma marginale* was identified from cattle. The sequences showed 100% identity with *A. marginale* strain Dawn (GenBank: CP006847), Gypsy Plains (GenBank: CP006846), Florida (GenBank: NR076579) and 99% identity with *A. centrale* strain Israel (GenBank: NR076686). *Anaplasma centrale* was also identified in cattle blood samples (9.4%) and showed 100% identity with *A. centrale* strain Israel (GenBank: NR076686) and 99% identity with *A. marginale* strain Dawn (GenBank: CP006847), Gypsy Plains (GenBank: CP006846) and Florida (GenBank: NR076579). From cattle sampled in the Sine Saloum, we obtained sequences of poor quality in 11/47 cattle. BLAST analysis showed that these sequences had the same homology (98–99%) and belonged either to *A. marginale* or *A. centrale.* We removed these poor sequences from further analysis. From sheep, cattle and goats, identical sequences were obtained. They showed 99% identity with *A. platys* stain ChieGuy88 (GenBank: KM021414) reported from French Guiana, *A. platys* strain Dog Gard1 (GenBank: KM021412) reported from France and *A. platys* strain ChieCal05 (GenBank: KM021425) reported from New Caledonia. Finally, from sheep, cattle and goats, another potentially new species was identified. The obtained sequences of this species were identical to each other and showed 93% identity with *A. marginale* strain Dawn (GenBank: CP006847), Gypsy Plains (GenBank: CP006846) and Florida (GenBank: NR076579), and 92% identity with *A. centrale* strain Israel (GenBank: NR076686) (Table 2).

From dogs, *A. platys* were found to infect 10/64 (15.6%) of dogs. The sequences were identical to each other and showed 99% homology with *A. platys* amplified from sheep, cattle and goats in the present study and 99% identity with *A. platys* stain ChieGuy88 (GenBank: KM021414), *A. platys* strain Dog Gard1 (GenBank: KM021412) and *A. platys* strain ChieCal05 (GenBank: KM021425). In addition, 12/64 (18.8%) of dogs were also infected with *Ehrlichia canis* that showed 100% with the sequences of *E. canis* amplified from the two *R. e. evertsi* in the present study and 99% identity with *E. canis* strain Jack (GenBank: NR076375).

**Fig. 3:**
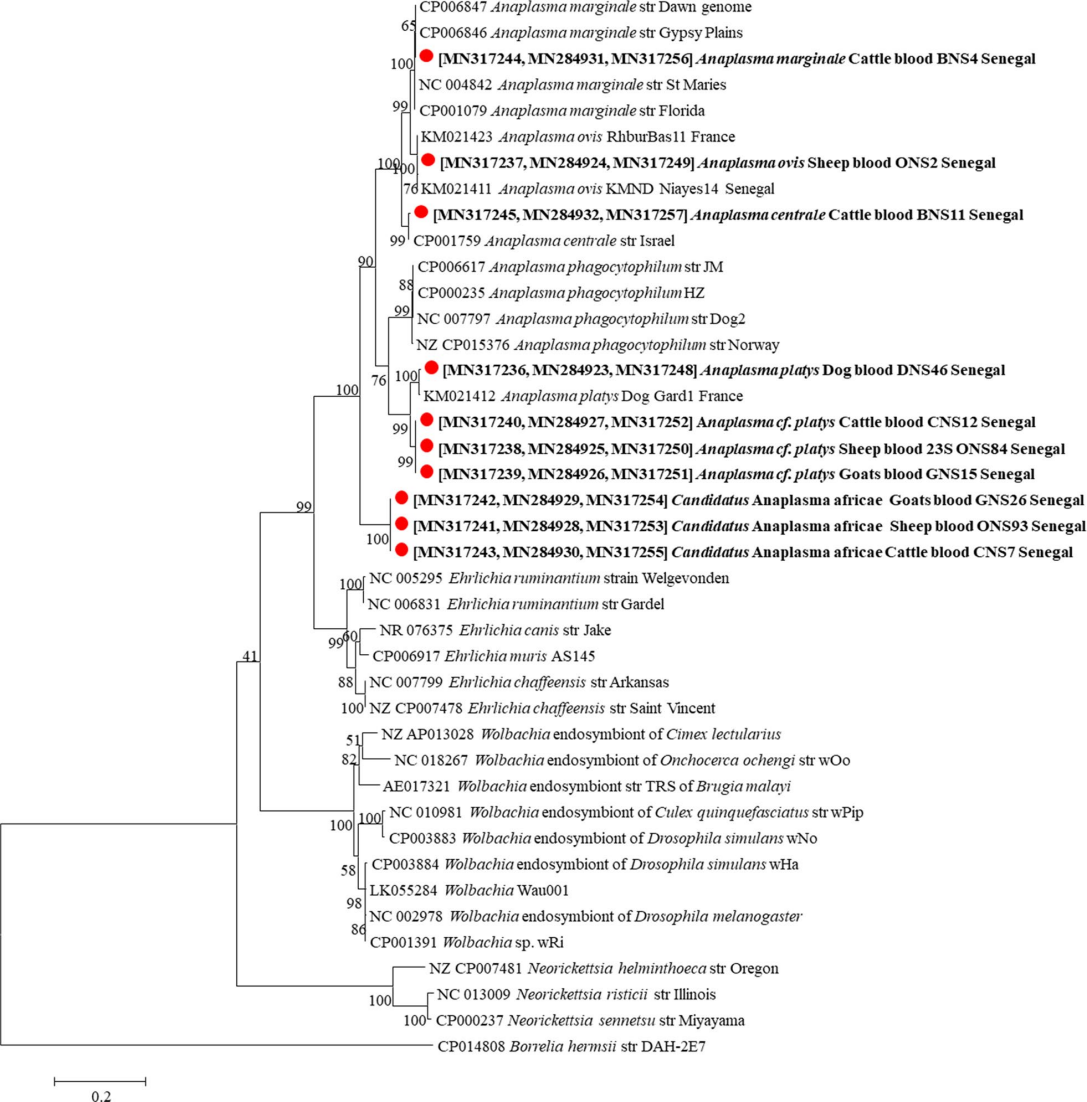
Phylogenetic tree showing the position of *A. ovis*, *A. marginale*, *Anaplasma* cf. *platys* and “*Ca.* Anaplasma africae” amplified from ruminates and *A. platys* amplified from dogs compared to other *Anaplasmataceae* species. Evolutionary analyses were conducted using MEGA7 [26]. The concatenated *23S* rRNA, *16S* rRNA and the *rpoB* genes of the *Anaplasma* spp. amplified in this study with other sequences of *Anaplasmataceae* species available from GenBank were aligned using CLUSTAL W implemented on BioEdit v.3 [25] (there were 1599 positions in the final dataset). The evolutionary history was inferred by using the maximum likelihood method based on theHasegawa–Kishino–Yano model. The percentage of trees in which the associated taxa clustered together is shown next to the branches. Initial tree for the heuristic search was obtained automatically by applying Neighbor-Join and BioNJ algorithms to a matrix of pairwise distances estimated using the maximum composite likelihood approach and then selecting the topology with superior log likelihood value. Statistical support for internal branches of the trees was evaluated by bootstrapping with 1000 iterations. A discrete gamma distribution was used to model evolutionary rate differences among sites [2 categories (+G, parameter = 0.3463)]. The analysis involved 41 nucleotide sequences. All positions containing gaps and missing data were excluded. The tree is drawn to scale, with branch lengths measured in the number of substitutions per site. The scale-bar represents a 20% nucleotide sequence divergence. Accession number for each concatenated sequence in the phylogenetic tree were provided for each species as [*23S* rRNA, *rpoB* and *16S* rRNA]

For further molecular identification targeting other genes for the family *Anaplasmataceae*, the two *R. e. evertsi* that harbored *E. canis* and for each different identified *Anaplasmataceae* species amplified by the primers targeting the *23S* rRNA gene, three randomly selected samples were chosen and the DNA was used to amplify a 828-bp long fragment of the *Anaplasmataceae* family *16S* rRNA, an *Anaplasma* spp. 525-bp long fragment of the RNA polymerase subunit beta (*rpoB*) gene and an *Ehrlichia* spp. 590-bp long fragment of the heat-shock protein (*groEL*) gene. The amplification followed by sequencing result showed that *A. ovis 16S* rRNA sequences amplified from sheep had 99% identity with *A. ovis* reported worldwide, whereas the *rpoB* sequences showed 100% identity with *A. ovis* strain RhburBas11 (GenBank: KX155495) reported from France and KMND Niayes 14 (GenBank: KX155494) reported from Senegal. *Anaplasma marginale 16S* rRNA sequences amplified from cattle showed 99% identity with multiple sequences of *A. marginale* reported from Uganda and with *A. marginale* strains Dawn (GenBank: CP006847) and Gypsy Plains (GenBank: CP006846). The *rpoB* sequences of this *A. marginale* strain showed 99% identity with *A. marginale* strain Dawn (GenBank: CP006847), Gypsy Plains (GenBank: CP006846) and Florida (GenBank: CP001079). The *16S* rRNA sequences of *A. centrale* showed 99% of identity with *A. centrale*, *A. ovis* and *A. marginale* reported worldwide. The *rpoB* gene sequence of *A. centrale* has 99% identity with *A. centrale* strain Israel (GenBank: CP001759) and 88% identity with *A. marginale* strain Dawn (GenBank: CP006847). The*16S* rRNA and *rpoB* sequences of *A. platys* amplified from ruminant were amplified from three samples each taken from sheep, goats and cattle. For each gene, the sequences were identical to each other and showed for the *16S* rRNA 99% identity with multiple uncultured *Anaplasma* sp., *A. platys* (GenBank: KY114935-EF139459), *A. platys* isolate A.pl. #87 (GenBank: JQ396431), “*Candidatus* Anaplasma camelii” clone Camel_38 (GenBank: KF843827) and 99% identity with *A. phagocytophilum* reported worldwide. The *rpoB* sequences showed 93% identity with *A. platys* strain Dog Gard1 (GenBank: KX155493) and 89% with *A. phagocytophilum* strain Norway variant2 (GenBank: CP015376). Sequence analysis of the three genes used in the present study revealed that this species is distinct from *A. platys* (99% for *23S* rRNA, 99% for *16S* rRNA and 93% for *rpoB*). Due to the absence of additional data on this *Anaplasma* sp. and the genetic relatedness to *A. platys*, we refer to this genotype here as *Anaplasma* cf*. platys*. A phylogenetic tree based on the concatenated *23S* rRNA, *16S* rRNA and *rpoB* genes showed that *Anaplasma* cf*. platys* form a separate and well-supported (bootstrap support of 99%) branch on the phylogenetic tree belonging to the cluster of *A. platys* (Fig. 3). The potentially new species identified from ruminants were also amplified from three samples taken from each ruminant species. The nine sequences from each gene (*16S* rRNA or *rpoB*) were identical to each other and showed for the *16S* rRNA 97% identity with multiple *A. phagocytophilum* reported worldwide, multiple *A. bovis* sequences reported from China and multiple sequences of uncharacterized *Anaplasma* spp. reported from China and Malaysia. The *rpoB* sequence of this species showed 79% with *A. centrale* strain Israel (CP001759), *A. phagocytophilum* strain Dog 2 (GenBank: CP006618), JM (GenBank: CP006617) and 78% with *A. marginale* strain Dawn (GenBank: CP006847) and Gypsy Plains (GenBank: CP006846). Sequence analysis of the concatenated *23S* rRNA, *16S* rRNA and *rpoB* genes revealed that this *Anaplasma* species was distinct from other *Anaplasmataceae* species considering the lower sequence identity (93% for *23S* rRNA, 97% for *16S* rRNA and 79% for *rpoB*). Because these potentially new species had not previously been reported, we propose the provisional name “*Candidatus* Anaplasma africae”. The phylogenetic tree showed that “*Candidatus* Anaplasma africae” form a separate and well-supported (bootstrap support of 100%) branch on the phylogenetic tree situated between *Anaplasma* spp. and *Ehrlichia* spp., albeit closer to the *Anaplasma* spp. clusters (Fig. 3).

**Fig. 4:**
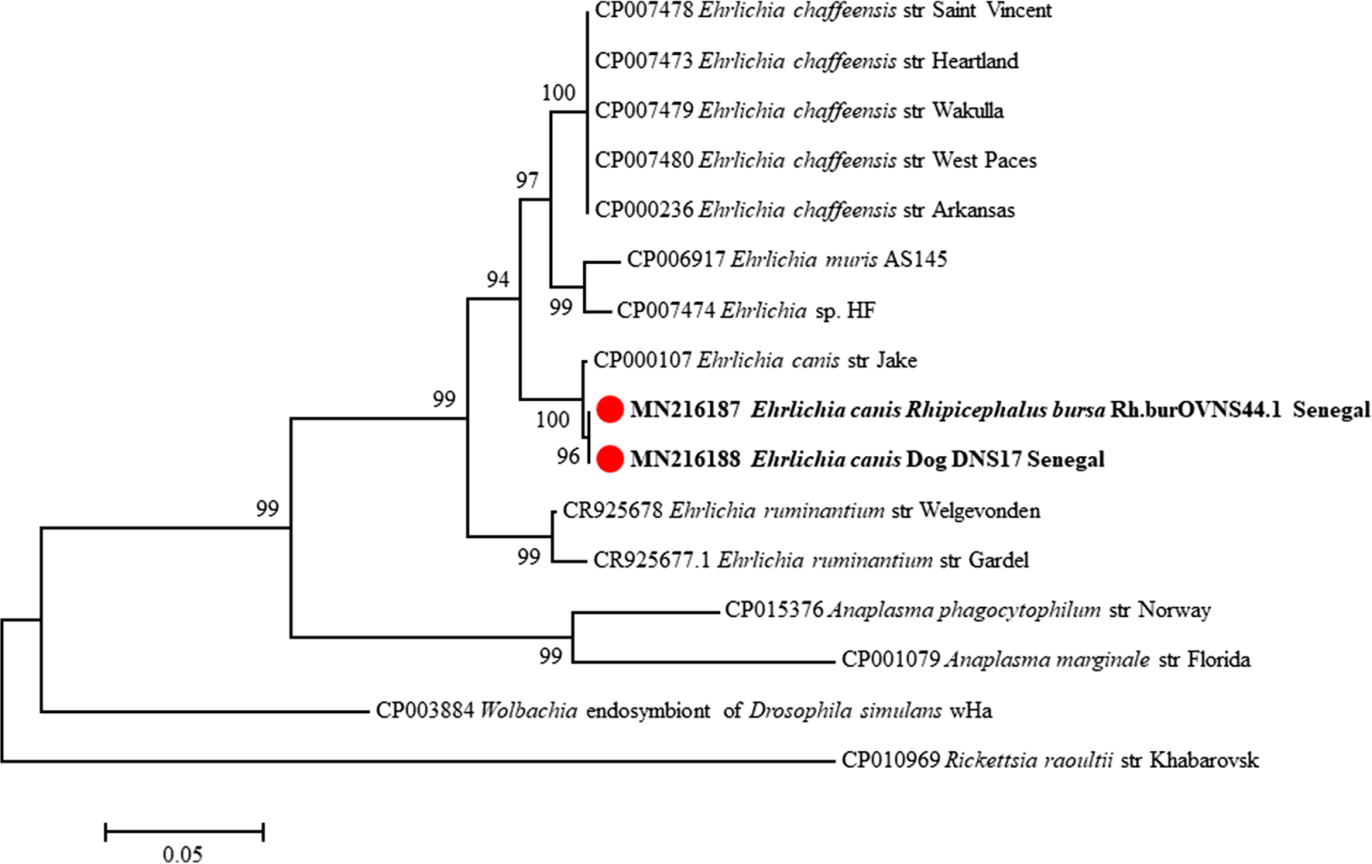
Phylogenetic tree showing the position of *E. canis* amplified from dogs and *Rhipicephalus bursa* ticks collected from sheep compared to other *Anaplasmataceae* species. Evolutionary analyses were conducted using MEGA7 [26]. The concatenated *23S* rRNA, *groEl* and *16S* rRNA genes of the *E. canis* amplified in this study with other sequences of *Anaplasmataceae* species available from GenBank were aligned using CLUSTAL W implemented on BioEdit v.3 [25] (there were 1818 positions in the final dataset). The evolutionary history was inferred by using the maximum likelihood method based on theHasegawa–Kishino–Yano model. The percentage of trees in which the associated taxa clustered together is shown next to the branches. Initial tree for the heuristic search was obtained automatically by applying Neighbor-Join and BioNJ algorithms to a matrix of pairwise distances estimated using the maximum composite likelihood (MCL) approach and then selecting the topology with superior log likelihood value. Statistical support for internal branches of the trees was evaluated by bootstrapping with 1000 iterations. A discrete gamma distribution was used to model evolutionary rate differences among sites [2 categories (+G, parameter = 0.4640)]. The analysis involved 41 nucleotide sequences. All positions containing gaps and missing data were excluded. The tree is drawn to scale, with branch lengths measured in the number of substitutions per site. The scale-bar represents a 5% nucleotide sequence divergence

The sequences of *A. platys 16S* rRNA amplified from dogs showed 99% identity with the sequences of *A. platys* amplified from ruminants in the present study and 99% with multiple sequences of *A. platys* reported worldwide. However, they also showed 99% identity with “*Candidatus* Anaplasma camelii” clone Camel_38 (GenBank: KF843827). The *rpoB* sequence showed 99% identity with *A. platys* strain Gard 1 (GenBank: KX155493) (Fig. 3). The *16S* rRNA sequences of *E. canis* amplified from dogs and the two *R. e. evertsi* ticks showed 99% identity with *E. canis* reported worldwide and 99% identity with “*E. ovina*” (GenBank: AF318946). The *groEL* sequences of this *E. canis* strain showed 99% with *E. canis* isolate D12E (GenBank: JN391407-JN391408) reported from the Philippines and *E. canis* strain Jake (GenBank: CP000107) reported from the USA (Fig. 4).

## Discussion

The clinical presentation of anaplasmosis depends on multiple factors including the *Anaplasmataceae* species, strain and host. Other factors associated with immune status and co-infections with other pathogens make the diagnosis a very challenging task. In addition, the economic impact, the zoonotic potential and the presence of multiple vectors associated with the transmission of these bacteria determine the need for accurate and direct laboratory tests [15]. Epidemiological data about the spread of bacteria belonging to the family *Anaplasmataceae* in addition to the identification of potential reservoir and vectors in the region will facilitate the interpretation of bacteriological results among the infected hosts. The present study summarizes entomological and epidemiological data of the prevalence of *Anaplasmataceae* species infecting animals and ticks in three regions of Senegal. In Keur Momar Sarr, five different tick species were collected from sheep, goats, cattle, horses, donkeys and dogs. Except for *R. bursa*, other ticks, namely *R. e. evertsi*, *Hy. rufipes*, *Hy. impeltatum* and *R. muhsamae*, had already been reported in Senegal and belong to the 33 known species in this country [27]. Walker et al. [20] states that the records of *R. bursa* from outside the Palaearctic region are linked to misidentification or accidental importation [20]. This species is usually recorded in the Mediterranean area, and some cold regions of Europe including the French Basque country [22] and Crimea [28]. In the present study, three engorged females removed from three horses were identified by molecular characterization as *R. bursa.* The occurrence of this species was very low 3/204 (1.5%). The data collected in the present study are insufficient to conclude or suggest an accidental introduction or for a possible establishment and/or the presence of foci of these ticks in the arid conditions of the northern region of Senegal.

The most abundant tick species found was *R. e. evertsi* (84.3%), collected from almost all animal species except from dogs. *Rhipicephalus e. evertsi* is an Afrotropical species. In West Africa, *R. e. evertsi* is absent on wild ungulates, suggesting that the species was introduced on domestic livestock from East Africa [20]. This species is the most common tick in southern Senegal [29]. In Africa, *R. e. evertsi* is present in a band extending roughly from 10–16°N and 11°W to 20°E, a region that receives between 400–1000 mm rainfall annually [20]. *Rhipicephalus e. evertsi* is known in Senegal as a potential vector of *Rickettsia africae*, *R. aeschlimannii*, *R. conorii* and *Coxiella burnetii* [27, 29, 30]. In our study, 2/176 (1.1%) *R. e. evertsi* collected from sheep were infected with *E. canis.* These two ticks were removed from the same sheep. To the best of our knowledge, this is the first identification of *E. canis* in *R. e. evertsi*. In previous studies, *A. platys*, *E. ruminantium* and *A. ovis* were also reported from *R. e. evertsi* in South Africa and Ethiopia [31–33]. However, to our knowledge, *E. canis* has never been reported from sheep. *Rhipicephalus e. evertsi* is a two-host tick species with larvae and nymph infesting the first host and adults infesting the second host [18]. Interestingly, *R. e. evertsi* was shown experimentally to transmit “*E. ovina*” to sheep by adult ticks. “*Ehrlichia ovina*” is a not a completely described *Ehrlichia* species but it is reported to infect domestic ruminants from Turkey and the Caribbean islands [34, 35]. The *16S* rRNA sequences of “*E. ovina*” (GenBank: AF318946) showed 100% identity with multiple *E. canis* sequences reported worldwide [36], whereas the *gltA* sequence (GenBank: KP719095) showed 99.9 % (two mismatches) identical to that of *E. canis* from Italy [34]. Nonetheless, stocks of “*E. ovina*” are not readily available and their taxonomic position needs to be analyzed for other genes including *groEL* [1] and *rpoB* [22]. These phylogenetic analyses are necessary to understand the validity of “*E. ovina*” as a species or to combine it with *E. canis*, as well as to confirm and consider *R. e. evertsi* as a competent vector for *E. canis*.

*Hyalomma rufipes* ticks were removed only from cattle and horses. This species represents 8.3% of the overall tick species collected. *Hyalomma rufipes* is most common in dry areas and distributed in almost all African countries [29]. This tick is found on a large variety of animals include wild mammals and birds and is considered an important vector of the Crimean-Congo hemorrhagic fever [37]. In Senegal, this tick has been identified as a host for *R. aeschlimannii* and *C. burnetii* [30]. In South Africa, *A. marginale* was found in these ticks [32, 38]. None of the *Hy. rufipes* tested here were infected by *Anaplasmataceae* species.

*Hyalomma impeltatum* was collected exclusively from horses at low occurrence (4.9%). In Senegal, *Hy. impeltatum* is more frequently encountered in cows than in sheep [29]. This Afrotropical tick was also reported infected by *R. aeschlimannii* [29] and interestingly by *Ehrlichia chaffensis* in Nigeria [39]. *Ehrlichia chaffensis* is an emerging bacteria in Africa, and until now, this species has been reported in three countries in Africa including Nigeria, Uganda and Cameroon [39, 40]. *Rhipicephalus sanguineus* and *Hy. impeltatum* seem to be potential vectors of *E. chaffensis* in Africa [39, 41]. In our study, none of the ten *Hy. impeltatum* collected were infected by an *Anaplasmataceae* species. However, *E. chaffensis* has never been reported from humans or other animals in Senegal. Finally, only two *R. muhsamae* (0.9%) were collected exclusively from dogs. *Rhipicephalus muhsamae* is an Afrotropical species found mainly in forest savanna mosaic and less abundant in tropical and subtropical moist broadleaf forest [18]. *Rickettsia conorii* has been detected in *R. mushamae* ticks removed from cattle in the Central African Republic [42]. None of the two *R. mushamae* collected were infected by *Anaplasmataceae* species.

Four hundred thirty-three blood samples were collected from different animals including sheep, goats, cattle, horses and dogs. The overall prevalence of *Anaplasmataceae* infection in the sampled animals was 41.1%. *Anaplasma ovis* was the common species found in sheep (55.9%). The infection prevalence found in the present study was higher compared to that previously reported in Senegal in 2013 (31.6%) [43]. Sheep develop persistent infections which allow them to become reservoirs of infection, explaining the high rate of infection by *A. ovis* in sheep [5]. The infection is usually subclinical and associated with hemolytic anemia. Cross-infection with other parasites or other stress conditions increase the severity of the infection [14]. Complication with other opportunistic diseases or stress conditions in sheep infected by *A. ovis* lead to the development of an acute disease phase characterized by fever, progressive anemia, icterus, weight loss, milk yield decrease and sometimes death [44]. In Africa, *A. ovis* has been reported from sheep in Ethiopia, South Africa, Tunisia [33, 44, 45] and from goats in Angola [46]. *Rhipicephalus e. evertsi* was previously reported as an important vector of *A. ovis* in Africa [37, 38]. However, none of the *R. e. evertsi* collected here was infected by *A. ovis*. *Anaplasma marginale* and *A. centrale* were identified in 19.4 and 8.1% of cattle, respectively. These two pathogens have never been reported in Senegal but have been reported from many other countries in Africa including Algeria, Angola, Botswana, Egypt, Ivory Coast, Kenya, Morocco, Mozambique, Nigeria, South Africa, Sudan, Tanzania, Tunisia, Uganda and Zambia [47–54]. The vectors of these two pathogens in Africa were ticks belonging to the genera *Rhipicephalus* and *Amblyomma* [5, 52]. The prevalence of these bacteria in cattle reported from the eastern and southern countries of Africa ranges between 32.1–100% [51]. Infection with *Anaplasma marginale* is associated with mild to severe anemia. Cattle infected with this bacterium developed various clinical signs including fever, decline in milk production, temporary infertility, and some animals developed an acute disease that manifested with gastrointestinal and neurological signs associated with the development of icterus seen during early convalescence. Mortality rates can reach 50–60% in adult cattle [5, 12]. *Anaplasma centrale* is less pathogenic and has been used as a live blood vaccine to protect against bovine anaplasmosis caused by *A. marginale* [55].

*Anaplasma* cf*. platys* found in this study was gentically close to the dog pathogen *A. platys.* Many genotypes close to *A. platys*, commonly named *Anaplasma platys*-like bacteria, were reported from animal families other than dogs [56]. *Anaplasma platys-*like is considered an emerging bacterium in many countries of the world. The same bacterium was reported previously from two sheep in Senegal [43]. In the present study, the prevalence found was 27.7% in goats, 22.6% in cattle and 19.8% in sheep. This species was initially characterized from ruminants in Italy [57]. Then, *Anaplasma* cf*. platys* (the commonly called *Anaplasma platys-*like) was reported from cattle in Algeria and Tunisia, from sheep in South Africa and goats in China [23, 45, 58, 59]. Recently this infection was also characterized from cats in Italy [56]. This species is described as a neutrophil-tropic *Anaplasma* sp. in ruminants and platelet-tropic in cats [57]. The strain identified here is genetically closest to the canine *A. platys* (nucleotide homology was 99% for *rrs* and *rrl*); however, the *rpoB* gene showed 93% identity with *A. platys*. *Anaplasma* cf*. platys* is located independently in a separate group in the phylogenetic tree based on the concatenated genes *rrl*, *rrs* and *rpoB* (Fig. 2).

The putative new species identified here, provisionally named “*Ca.* Anaplasma africae”, has genetic features which are different from all other species of the genus *Anaplasma*. A phylogenetic tree based on the concatenated *rrl*, *rrs* and *rpoB* genes showed that “*Ca.* Anaplasma africae” forms a separate branch on the phylogenetic tree situated between *Anaplasma* and *Ehrlichia*, albeit closer to the *Anaplasma* clusters (Fig. 2). The prevalence of this species is low in goats, cattle and sheep, 10.3%, 8.1% and 3.7%, respectively. The importance of *Anaplasma* cf*. platys* and “*Ca.* Anaplasma africae” in ruminants has to be elucidated.

*Ehrlichia canis* and *A. platys* were identified exclusively from dogs sampled in the Keur Momar Sarr villages. None of the dogs sampled in the Casamance region were found positive. In dogs from Keur Momar Sarr, the prevalence observed was 18.8% for *E. canis* and 15.6% for *A. platys.* The prevalence of *E. canis* reported in the present study was low compared to previous reports in dogs sampled in a kennel in Dakar, Senegal (53%). However, the prevalence of *A. platys* was higher than what was reported in the same study (5.9%) [60]. *Ehrlichia canis* infection in dogs induces monocytotropic ehrlichiosis and results in various signs depending on the disease stages [61], whereas *A. platys* induces a recurrent thrombocytopenia that can be resolved in the absence of other infecting agents or complicating factors [62, 63]. The prevalence to *E. canis* and *A. platys* in Africa is poorly known; however, these two species are apparently ubiquitous throughout the African area where the *R. sanguineus* tick group is spread [17, 64].

Nonetheless, dogs from the Central African Republic and Reunion Island were all negative for *E. canis* [65]. *Rhipicephalus sanguineus* (*s.l.*) in the tropical and subtropical region are active throughout the year and apparently have no seasonality [66]. In our study, two *R. mushamae* ticks were collected from two dogs. To the best of our knowledge, *A. platys* has never been associated with *R. mushamae. Ehrlichia canis* has been reported once from *R. mushamae* in Mali [17]. In Africa, from tick species other than *R. sanguineus* (*s.l.*), *A. platys* was amplified from *R. e. evertsi* salivary glands in South Africa [32] and *R. camicasi* in Kenya and Ivory Coast [66].

None of the 64 horses tested were found positive for an *Anaplasmataceae* infection. In addition, none of the *Anaplasmataceae* species identified from ruminants and dogs were identified in ticks, except for *E. canis* identified in dogs and *R. e. evertsi* collected from sheep. *Anaplasma* cf*. platys* and “*Ca.* Anaplasma africae” were found in sheep, goats and cattle. Despite the fact that *R. e. evertsi* were collected from different ruminant animals, none of these ticks were found positive for any *Anaplasmataceae* species. *Anaplasma phagocytophilum* was also not found in the present study. *Anaplasma phagocytophilum* has already been identified in sheep in Senegal, although all of its known vectors are absent in sub-Saharan Africa [43]. Evidence of the presence of *A. phagocytophilum* in Africa has been reported from Morocco, Algeria and Tunisia [23, 54, 67], where *Ixodes ricinus* has been identified in some foci in the northern regions of these countries [18]. This infection has also been reported from wildlife in Zimbabwe and South Africa [68, 69], and from some African ticks such as *Ambyomma flavomaculatum* collected from two different specimens of lizard (*Varanus exanthematicus*) imported to Poland from Ghana [70], from *A. cohaerens*, *A. lepidum* and *A. variegatum* in Ethiopia [71, 72] and from *Hy. marginatum* in Tunisia [53]. In our study, none of the five tick species were found to be infected by *A. phagocytophilum*, suggesting the implication of other tick species in the epidemiology of *A. phagocytophilum* in sub-Saharan Africa.

## Conclusions

The present study aimed to identify *Anaplasmataceae* species infecting animals and ticks and determine a possible epidemiological *Anaplasmataceae*-host-tick connection. The present work indicates that ruminants and dogs in the northern and central areas of Senegal are a reservoir for multiple *Anaplasmataceae* species. The prevalence of *A. ovis* and *A. marginale* was high in sheep and cattle, respectively. Molecular analysis revealed an interesting diversity of *Anaplasmataceae* infections in ruminants and dogs including a potentially new species infecting ruminants. However, except for *E. canis*, none of the other *Anaplasma* spp. found in the present study was amplified from ticks. Nevertheless, further studies are needed to ascertain the *Anaplasmataceae-*host-vector connections in sub-Saharan Africa as well as to decipher the zoonotic potential of newly identified genotypes and their significance for animals and public health.

## Data Availability

All data are available from the corresponding author upon reasonable request. Representative sequences were submitted to the GenBank database under the following accession numbers. The *23S* rRNA gene sequences: *A. platys* DNS46 [MN317236], *A. ovis* SNS2 [MN317237], *A.* cf. *platys* from sheep (SNS84), goats (GNS15) and cattle (CNS12) [MN317238, MN317239 and MN317240, respectively], “*Ca.* Anaplasma africae” from sheep (SNS93), goats (GNS26) and cattle (CNS7) [MN317241, MN317242 and MN317243, respectively], *A. marginale* CNS5 [MN317244], *A. centrale* CNS11 [MN317245], *E. canis* from *R. bursa* [MN317246] and dogs [MN317247]. The *rpoB* gene sequences: *A. platys* DNS46 [MN284923], *A. ovis* SNS2 [MN284924], *A.* cf. *platys* from sheep (SNS84), goats (GNS15) and cattle (CNS12) [MN284925, MN284926 and MN284927, respectively], “*Ca.* Anaplasma africae” from sheep (SNS93), goats (GNS26) and cattle (CNS7) [MN284928, MN284929 and MN284930, respectively], *A. marginale* CNS5 [MN284931], *A. centrale* CNS1 [MN284932]. The *GroEL* gene sequences: *E. canis* from dogs [MN216188], *E. canis* from *R. bursa* [MN216187]. The *16S* rRNA gene sequences: *A. platys* DNS46 [MN317248], *A. ovis* SNS2 [MN317249], *A.* cf. *platys* from sheep (SNS84), goats (GNS15) and cattle (CNS12) [MN317250, MN317251 and MN317252, respectively], “*Ca.* Anaplasma africae” from sheep (SNS93), goats (GNS26) and cattle (CNS7) [MN317253, MN317254 and MN317255, respectively], *A. marginale* CNS5 [MN317256], *A. centrale* CNS11 [MN317257], *E. canis* from *R. bursa* [MN317258] and dogs [MN317259]. The tick *12S* rRNA gene sequences: *R. evertsi evertsi* from sheep, equines, donkeys and cattle [MN315378, MN315379, MN315380 and MN315381, respectively]; *H. rufipes* from equines and cattle [MN315382 and MN315383, respectively]; *H. impeltatum* from equines [MN315384]; *R. bursa* from equines [MN315385]; and *R. muhsamae* from dogs [MN315386].

## Abbreviations

Ap-ha: *Anaplasma phagocytophilum* strain human-active
Ap-v1: *Anaplasma phagocytophilum* variant 1
SNP: single-nucleotide polymorphism
